# An Experimental Investigation into Promoting Mental Health Service Use on Social Media: Effects of Source and Comments

**DOI:** 10.3390/ijerph17217898

**Published:** 2020-10-28

**Authors:** Zhaomeng Niu, Lun Hu, David C. Jeong, Jared Brickman, Jerod L. Stapleton

**Affiliations:** 1 Rutgers Cancer Institute of New Jersey, New Brunswick, NJ 08901, USA; 2 Xinjiang Technical Institute of Physics and Chemistry, Chinese Academy of Sciences, Urumqi 830011, China; 3 Department of Communication, Santa Clara University, Santa Clara, CA 95053, USA; dcjeong@scu.edu; 4 Carnegie Dartlet, Portland, OR 97201, USA; jsbrickman@wsu.edu; 5 College of Public Health, University of Kentucky, Lexington, KY 40506, USA; jerod.stapleton@uky.edu

**Keywords:** social media, Facebook, mental health, source credibility, valence of comments, affective trust

## Abstract

Mental health is an increasingly prevalent topic of public interest, but remains a complex area requiring focused research that must account for negative perceptions surrounding mental health issues. The current work explores the roles of social media information source credibility and valence of social media comments on health outcomes in such a mental health context. We used a 2 (message source: professional vs. layperson) × 3 (valence of comments: positive vs. negative vs. mixed) online experiment to examine the effects of source and valence of comments on trust, attitudes and intentions related to mental health information and services among 422 undergraduate students. Results supported the hypothesized model in which source influenced cognitive trust while comments influenced affective trust. Cognitive and affective trust both impacted attitudes towards mental health information which encourages the intention to share such information on social media. Additionally, affective trust impacted attitudes towards mental services which influenced intentions to seek them out. Source and valence of comments on social media impact different behavioral intentions regarding the use of mental health services. This study provides insights for future social media campaigns promoting mental health service use.

## 1. Introduction

Nearly 1 in 5 of all adults in the United States, approximately 46.6 million people, experienced mental illness in 2017 [1]. Young adults have the highest prevalence of any mental illness (25.8%) [1], making it one of the greatest health concerns for this population. While mental health or mental illness may refer to a range of disorders and conditions (e.g., anxiety, depression, schizophrenia), it is often incorrectly perceived as less important than physical health due to its lack of visual symptoms [2]. The consequences of lack of treatment of mental illness include serious health concerns, such as high risk for chronic medical conditions and suicide. However, only 41% of those with a mental illness have sought treatment [3]. Low rates of utilization of mental health services may be attributed to an unwillingness to seek treatment due to distrust or negative attitudes towards such services.

Promisingly, positive information about mental health delivered on social media has been shown to reduce negative perceptions and change unfavorable attitudes towards mental health counseling [4]. Engaging social media users and sharing personal stories about mental health could help spread information and change attitudes about seeking treatment [5]. However, health communication on social media may also come from sources perceived to have questionable credibility and may be accompanied with negative comments from other social media users [6]. Therefore, it is important to understand how these different sources and the valence of comments influence how users process mental health information online and how such information impacts their users’ perceptions related to health information.

One critical concern in the current social media landscape is the credibility of online information [7,8]. People tend to judge credibility based on the perceived information source and prior research on source credibility has primarily focused on the impact of cognitive trust (perceived credibility of the information) on attitudes and intentions [9,10]. Affective trust, the emotional trust placed in information, can also impact audience attitudes and intentions [11]. This study is unique in its investigation of the differences between the effects of cognitive and affective trust on attitudes and behavioral intentions related to mental health services.

Perceptions of mental health issues may also be influenced by comments shared on social media. A key deterrent to seeking treatment for mental health issues is negative perception [6,12], which may stem in part from negative social commentary [13,14]. However, the additivity hypothesis [15] posits that comments that are consistent with a message could boost its persuasive effects, indicating that comments that support using mental health services could reinforce the message. No empirical study has investigated how the valence of user comments to social media messages, defined as the positive or negative emotional tone of a comment, impacts the persuasive effect of social media content regarding mental health services.

Given that cues on social media such as sources of content and user comments on such content may together impact viewers’ trust and perceptions of social media messages, the current study aimed to investigate the impact of sources (i.e., layperson and professional sources), and the valence of user comments on individuals’ trust (i.e., cognitive trust and affective trust) and health-related perceptions (i.e., attitudes and behavioral intentions) in the context of mental health as a communication process.

### 1.1. Mental Health and Social Media

While mental health or mental illness may refer to a range of disorders and conditions (e.g., anxiety, depression, schizophrenia), it is often incorrectly perceived as less severe than physical illness due to its lack of visual symptoms [2]. In fact, 18% [3] to 25% [16] percent of Americans suffer from a mental illness, making it the most prevalent national health issue. Perhaps even more alarmingly, only 41% of those with a mental illness have sought treatment [3], which may be attributed to a lack of awareness or an unwillingness to seek treatment. Specifically, reasons for not seeking mental health treatment are often tied to negative social commentary, which may stem from inaccurate public knowledge [13] and is often experienced by youth [14].

Promisingly, there are signs that mental health interventions on social media have reduced negative perceptions and are changing unfavorable attitudes toward mental health counseling [4] in a general audience, which suggests that engagement with user-generated content and shared articles about mental health could help spread information and change attitudes about seeking treatment [5]. The current study attempts to contribute to the ongoing mental health discussion on social media with an added emphasis on information source credibility and the shaping influence of others’ opinions.

### 1.2. The MAIN Model and Cues

Given the ease with which users may edit and publish information online without a rigorous gatekeeping process, concerns about the credibility of such information have been well-documented [17,18]. Online users typically need to utilize cues that aid their evaluation of the credibility of the content before taking actions based on the information (e.g., to form attitudes). The Modality Agency Interactivity Navigability (MAIN) model of digital media [19] explores the impact of technologically afforded heuristics on users’ judgment of the credibility of online information.

Social media platforms such as Facebook, and e-commerce sites such as Amazon have digital features and cues (e.g., “likes,” comments, ratings) indicating endorsement or disagreement with a message or a product. Such comments and “likes” comprise a critical feature in modern communication technology research as they have the potential to trigger a heuristic, where users tend to construct opinions regarding online content based on other users’ opinions (e.g., “*If others think that something is good, then I should, too*” [19]). Thus, the valence of user comments plays an important role in constructing perceptions toward social media content.

Closely related to endorsement, online users also often rely on authority cues about an information source to make judgments on online information [19]. This is particularly significant in the case of health information, which may come from many different sources that range in level of expertise and reliability. For instance, participants have reported different levels of perceived credibility and of behavioral intention of health information, depending on whether they were told the information was from a layperson source or a professional source [10]. In sum, social media cues such as the valence of user comments and the perceived authority of an information source are critical factors in understanding how people process health information regarding information credibility, attitudes, and intentions on social media.

Although source credibility and message valence on social media have been explored in previous health communication research, less is known about the impact of the valence of user comments on social media on shaping individuals’ opinions. Further, relatively little research has focused on how different types of cues in a social media environment work together in influencing individuals’ perceptions, as a communication process. The current study aimed to evaluate the impact of two types of cues, valence of comments and source type, on health information credibility, attitudes, and intentions as a communication process on social media.

### 1.3. Dimensions of Trust, Cues and Cognitive Perceptions

Based on prior work in psychology, e-commerce, advertising, and marketing, the concept of information trust may be split into two dimensions, cognitive trust and affective trust [20,21]. Although different items might be used to measure these two types of trust across different researchers [22], it is acknowledged that cognitive trust is logic-driven and affective trust is emotion-driven [11]. Cognitive trust is grounded in individuals’ rational assessment based on “evidence of trustworthiness” and is usually measured with objective adjectives such as “reliable” and “accurate” [23] and is thus similar to the concept of “perceived credibility”. In contrast, affective trust refers to how one feels about such trust attributes as warmth and affection when interacting with an objective, instead of reasoning and logic [24,25]. Affective trust is generated based on feelings [11], and commonly reflects positive and likable feelings [26]. Thus, it is usually measured via feeling and affections. Previous research [23,27] has operationalized “affective trust” from an emotional perspective with adjectives that describe feelings such as “likable” and “warm”.

Source can impact users’ cognitive trust of online information. Prior work on source credibility of health information has suggested that professional sources (i.e., authority cues) are perceived to be more credible than layperson sources [10]. Due to the user-generated nature of social media, which is different from traditional media, and the fact that anyone could be an information source, it is important to investigate the effects of source types of social media message on individuals’ perceptions of mental health, which is certainly a sensitive but critical issue to address in contemporary health communication.

In addition, there is evidence that suggests user comments may be more influential than message source in forming cognitive perceptions towards health information, such as attitudes and behavioral intentions [28,29]. For example, Ahn [30] found that positive comments that support the content yielded more favorable feelings toward the content than negative comments. Further, online news with comments supporting one opinion/direction have been observed to influence perceptions more strongly than those with no comments or mixed comments [31]. More empirical research is needed to examine the relationship between the valence of comments and cognitive perceptions regarding health content.

Though prior research mainly focuses on how the above two types of trust are formed through different types of information process [23], less is known about how these two types of trust influence attitudes and behavioral intentions under different health contexts. Individuals’ cognitive trust is positively related to health perceptions [32]. Marton and Choo [33] indicated that cognitive trust positively influences participants’ attitudes toward online health information, which is a positive and substantial predictor of intention to use the health information online [34]. Affective trust was found to account for variances in persuasion and influenced behavioral beliefs and attitudes toward health messages [23]. Therefore, affective trust may influence attitudes about sharing the social media messages with others, and/or lead to a greater intention to use mental health services.

The current study aims to measure both attitudes toward health information (Facebook posts) and attitudes toward mental health services along with intentions to share health information on social media and intention to use mental health services. Based on previous evidence, different types of trust may influence attitudes toward information or attitudes towards the object or behavior described in the information [35] and the Theory of Planned Behavior [36] posits that attitudes will influence relevant behavioral intentions. Therefore, trust in health information could impact different health attitudes and intentions.

### 1.4. Research Questions and Hypotheses

Based on the literature reviewed, the following hypotheses and research questions were developed:

**Hypothesis** **1** **(H1).** *Posts from a professional source will be perceived with greater cognitive trust than posts from a layperson source.*

**Hypothesis** **2** **(H2).** *Posts with positive comments will be perceived with greater affective trust than those with mixed or negative comments.*

**Hypothesis** **3** **(H3).** *(a) Cognitive trust and (b) affective trust in a Facebook post will be positively associated with attitudes towards Facebook posts about mental health information.*

**Hypothesis** **4** **(H4).** *(a) Cognitive trust and (b) affective trust in a Facebook post will be positively associated with attitudes towards mental health services.*

**Hypothesis** **5** **(H5).** *Attitudes towards Facebook posts about mental health information will be positively associated with intentions to (a) use mental health services, and (b) share the Facebook posts about mental health information.*

**Hypothesis** **6** **(H6).** *Attitudes towards mental health services will be positively associated with intentions to (a) use mental health services, and (b) share the Facebook posts about mental health information.*

**Figure 1:**
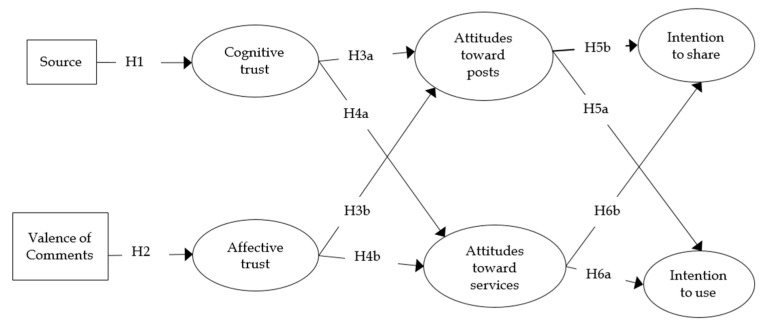
Conceptual Model of Source and Valence of Comments on Behavioral Intentions.

A conceptual model was proposed based on the theoretical relationships in Figure 1. Structural equation modeling (SEM) was used to examine the study hypotheses, as it is useful to understand health communication as a complex multivariate phenomenon rather than an isolated incidence [37,38].

## 2. Methods

### 2.1. Design Overview

Our experiment employed a between-subjects 2 (source: professional vs. layperson) × 3 (comments: positive, mixed or negative) factorial design in an online experiment. We evaluated the effects of different social media sources and the valence of comments on cognitive trust, affective trust, attitudes toward social media posts on health information, attitudes toward mental health services, behavioral intention to share mental health information on social media, and behavioral intention to use mental health services in the future.

### 2.2. Participants and Procedure

A total of 425 undergraduate students were recruited to participate in the online experiment on a participatory online system called Sona, incentivized by extra credit. We used a general student sample rather than limiting the study to students who were undergoing serious mental health issues. Since college students are heavy social media users and tend to experience a variety of mental issues such as depression and stress [39], a student sample is appropriate for the current study in order for researchers to design social media campaigns and preventions which promote mental health service use among those who may potentially suffer or will suffer from mental health problems in the future. Participants who signed up received a link to an online study and were randomly assigned to one of six experimental conditions. Subjects in each condition viewed three mock Facebook posts (and attached comments) and were given at least 60 s to view each post. After viewing all the mock Facebook posts, the participants completed a questionnaire about what they had viewed, as well as demographic questions. The study was approved by the university’s Institutional Review Board (WSU IRB # 15136).

### 2.3. Experimental Treatment Conditions

**Figure 2:**
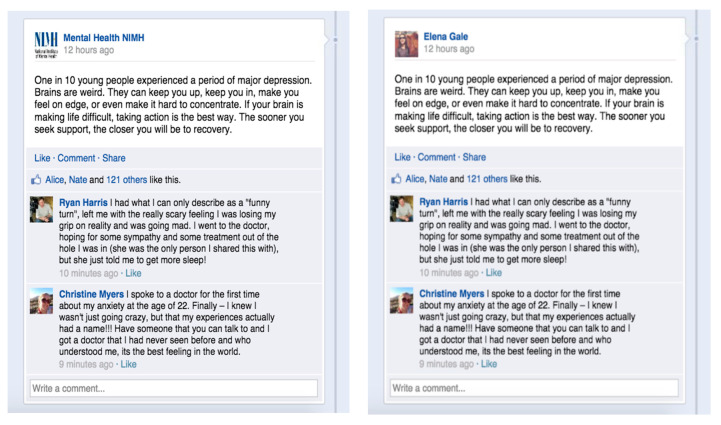
Professional Source 1 and Mixed Comments 1^a^ vs. Layperson Source 1 and Mixed Comments 1. ^a^ Each condition has three sets of screenshots. Professional source 1 and layperson source 1 have the same content except for account name and profile picture.

**Figure 3:**
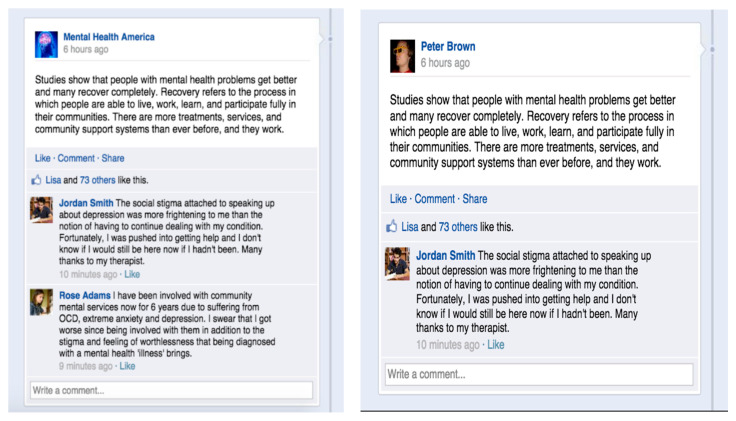
Professional Source 2 and Mixed Comments 2 vs. Layperson Source 2^a^ and Positive Comments 2^b^. ^a^ Layperson source 2 and professional source 2 have the same content except for account name and profile picture. ^b^ Layperson source 2 *×* negative comment 2 and Layperson source 2 × positive comment 2 only differ in the comment section.

A total of 18 mock Facebook posts were constructed through Facebook post generating tools by the researchers (3 per condition) and the users in the screenshots were not real users. While each of the six conditions varied according to source, comments and the number of likes on the post, the content of the posts remained the same. The sources for the three layperson conditions were named “Amy Jones,” “Peter Brown,” and “Elena Gale” (e.g., Figure 2 and Figure 3), and the source for the 3 professional conditions were named “Mental Health Professional,” “Mental Health America,” and “Mental Health NIMH (National Institute of Mental Health” (e.g., Figure 2 and Figure 3). In addition to names, professional and layperson sources also varied in profile pictures (See Figure 2). We also included two comments on each post from both a male and a female “user” to reduce potential gender biases in the mixed comments condition (e.g., Figure 2). The positive comments and negative conditions had the same content as the mixed comments condition except that the comments are purely positive or negative (e.g., Figure 3). The Facebook post content was derived from the website of the NIMH (National Institute of Mental Health). Both negative and positive comments were taken from actual online comments on mental health services.

### 2.4. Measurements

#### 2.4.1. Manipulation Checks

At the end of the survey, we conducted two manipulation checks: (1) We conducted a manipulation check for the source of the Facebook posts by asking participants to indicate the extent to which they deemed the sources to be professional on a 5-point Likert scale ranging from 1 (“*very unprofessional*”) to 5 (“*very professional*”); (2) We conducted a manipulation check on the positive and negative valence of the comments by asking participants to indicate how positive or negative they felt about the comments on a scale from 1 (“*very negative*”) to 5 (“*very positive*”). Two independent-sample t-tests were used to compare the means of different groups of sources and comments. For both tests, the assumption of homogeneity of variance was not violated. Participants who viewed positive comments (*M* = 3.84, *SD* = 0.78, *n* = 148) indicated that the Facebook comments were significantly more positive than those who viewed negative comments (*M* = 2.24, *SD* = 0.75), *n* = 141), *t* (287) = 17.74, *p* < 0.001. Further, participants in the professional source group (*M* = 3.48, *SD* = 0.90) indicated that the source was significantly more professional than those in the layperson source group (*M* = 2.91, *SD* = 0.88), *t* (422) = −6.65, *p* < 0.001. As such, both manipulations (positive, negative; professional, layperson) were confirmed.

#### 2.4.2. Mediating Variables

Perceived credibility (cognitive trust) was measured using four items adapted from prior work [27,40] indicating whether the Facebook posts were “*accurate*”, “*reliable*”, “*credible*”, and “*believable*” on a 10-point Likert scale ranging from 1 (“*not at all*”) to 10 (“*extremely*”) (α = 0.88, *M* = 5.48, *SD* = 1.72).

Affective trust was measured by four items obtained from Koh and Sundar’s [27] and Kim and Sundar. Four 10-point Likert items ranging from 1 (“*not at all*”) to 10 (“*extremely*”) were used to measure whether the Facebook posts were “*likable*”, “*interested in my well-being*”, “*empathetic”,* and “*warm*” (*α* = 0.87, *M* = 5.49, *SD* = 1.75).

Attitude toward Facebook posts containing mental health information [41] was measured by seven items (*α* = 0.94, *M* = 4.57, *SD* = 1.19) asking whether the Facebook posts about mental health information were “*bad*” or “*good*,” “*unhelpful*” or “*helpful*,” “*unenjoyable*” or “*enjoyable*,” “*harmful*” or “*beneficial*,” “*worthless*” or “*valuable*,” “*foolish*” or “*wise*,” and “*not useful*” or “*useful*” on a 7-point Likert scale items, ranging from 1 to 7.

Similarly, attitude toward mental health services was measured using seven items as above (*α* = 0.97, *M* = 5.60, *SD* = 1.21).

#### 2.4.3. Dependent Variables

Intention to use mental health services was adapted from prior work on behavioral intention [41,42]. Three 5-point Likert-type items were used to measure respondents’ degree of agreement related to intentions to use mental health services if they had the need (*α* = 0.93, *M* = 3.79, *SD* = 0.85).

Intention to share mental health information was based on Hu and Sundar’s [10] adapted multidimensional scale measuring behavioral intention. Respondents indicated their degree of agreement regarding three 5-point Likert-type items about their behavioral intention to recommend the information to others such as “*I will forward this Facebook message to my online acquaintances*” (*α* = 0.85, *M* = 2.45, *SD* = 1.00).

### 2.5. Data Analysis

Since it is useful to understand health communication as a complex multivariate phenomenon rather than an isolated incidence [37,38], SEM was used to examine the mediating relationships as a communication process with Mplus Version 7.11 (Muthén & Muthén, Los Angeles, CA, USA) [43]. The analysis controlled for gender and race due to their potential influences on college students’ mental health. Female students may experience higher level of depression [39] and ethnic minority students are sometimes more likely to experience stress on campus [44]. ANOVA analysis was used in SPSS 24.0 (IBM Corp., Armonk, NY, USA) [45] for post-hoc comparison.

## 3. Results

### 3.1. Sample

In total, 425 undergraduate students were recruited and three incomplete responses were dropped. The final analytic sample was 422. Participants age ranged from 18 to 26 years old (*M* = 19.83, *SD* = 1.42), two-thirds of the sample was female (62.2%), and a majority identified as White (70.4%). Participants also reported being Hispanic (7.5%), African American (5.9%), and Asian/Pacific Islander and other (14.2%). Descriptive characters of the variables under study are shown in Table 1.

### 3.2. Results of SEM

**Figure 4:**
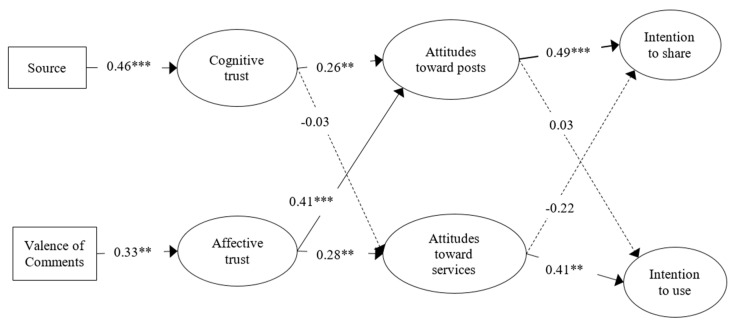
Model of Source and Valence of Comments on Behavioral Intentions. The model includes effects of control variables, which are not displayed. Indicators of each latent variable are not displayed. Dashed lines indicate non-significant paths. ** *p* < 0.01. *** *p* < 0.001.

The values of root mean squared error of approximation (RMSEA) and Comparative Fit Index (CFI) indicated a good model fit (Table 2) and the results of each path are shown in Figure 4. According to the results of the SEM analysis, race/ethnicity was associated with sharing intention and white participants were more likely to share mental health posts on Facebook (*p* < 0.05).

Posts from a professional source had a significant positive influence on cognitive trust (*β* = 0.46, *p* < 0.001), thus supporting H1.

The valence of comments had a significant effect on affective trust (*β* = 0. 33, *p* < 0.01). Since there were three levels of comments, we conducted an additional one-way ANOVA test to further examine the associations between the valence of comments and affective trust. Based on the results of the post-hoc test of the one-way ANOVA, the respondents who viewed positive comments (*M* = 6.08, *SD* = 1.86) reported higher affective trust about mental health services information than those who viewed mixed (*M* = 5.34, *SD* = 1.55, *p* < 0.001) or negative comments (*M* = 5.02, *SD* = 1.63, *p* < 0.001), thus H2 was supported.

Furthermore, higher cognitive trust (*β* = 0.26, *p* < 0.001) and affective trust (*β* = 0.41, *p*< 0.001) in a Facebook post were significantly associated with more positive attitudes towards Facebook posts, thus supporting H3a and H3b.

Only affective trust had a significant effect on attitude towards mental health services (*β* = 0.28, *p* < 0.001), thus H4b was supported while H4a was not.

Attitude towards Facebook posts led to greater intention to share the health information (*β* = 0.49, *p* < 0.001), but had no effect on intention to use mental health services. Therefore, H5a was not supported while H5b was supported.

Attitudes towards mental health services had a significant effect on intention to use mental health services (*β* = 0.41, *p* < 0.001), but did not have a significant effect on intention to share a post about mental health services; thus, H6a was supported while H6b was not.

## 4. Discussion

The present study examined the effects of two types of interface cues on social media, different types of sources and valence of comments, on behavioral intentions about using mental health services and sharing mental health related information on media. The results of our analysis indicate three sets of primary findings: (1) first, mental health posts delivered by professional sources were deemed as credible sources of information, while comments demonstrating positive or negative valence were associated with emotion-driven affective trust; (2) both cognitive and affective forms of trust were positively associated with one’s attitudes towards the mental health information delivered in the Facebook posts, which in turn promoted in individuals a greater intention to share the mental health information on social media; (3) only elevated affective trust positively impacted one’s attitudes towards mental health services, which in turn promoted in individuals a greater intention to actively use mental health services.

Elaborating on the above, we found that source cues affect users’ attitudes through cognitive trust in posts containing health information. If the source was perceived as more professional, respondents were more likely to have higher cognitive trust in the social media post that is shown to them. The effect of cognitive trust on behavioral intention to share Facebook posts about mental health was fully mediated by the level of attitude toward mental health posts. The professional source cues led to a rational and logical judgment of the health information on social media sites, which could result in a rational evaluation and favorable attitude toward information about mental health posts in the future. Moreover, the more favorable attitude the individual has, the more likely he or she will engage in sharing mental health counseling related information on his or her online social networks.

Additionally, we found strong evidence supporting that different types of social media comments significantly impact affective trust among viewers. More specifically, positively-valenced comments indicating support or agreement were associated with greater affective trust compared to negatively- or mixed-valenced comments. Further, we found that affective trust in posts about health information directly influenced attitudes towards mental health posts as well as attitudes toward mental health services, and influenced different intentions. More favorable attitudes towards mental health services were associated with greater intentions to use mental health services in the future. This finding suggests that positive comments towards mental health services on social media can help combat the negative perceptions around mental health on social media through their influences on affective trust [12,46].

The current work presents three primary contributions. First, as mental health is a topic not yet covered in the credibility and message valence literature, the present work offers new insights into this topic. Second, the current study sheds light on understanding the theoretical relationships between cues on social media and cognitive perceptions as a complex communication process. Finally, healthcare professionals can learn the importance of comments on social media in the comment section, in addition to credibly-sourced posts, in social media interventions.

### 4.1. Health Applications

Social media platforms are ubiquitous and offer channels for advertising, marketing and information seeking. It is critical to understand which technological features (e.g., comments) on social media sites have effects on health related cognitive, attitudinal, and behavioral outcomes [47]. The current study identified the mechanisms of health information processing on social media influenced by information source and valence of comments and suggests that different types of sources on social media sites may directly influence perceived credibility of health information, which in turn impacts attitudes toward the information as well as behavioral intention to share the mental health information with their social network.

We found a link between valence of comments and intention to use mental health services through affective trust and attitudes towards mental health services, which is critical for mental health research. Compared to sharing information online, seeking mental health services is arguably a more crucial behavior to directly impact positive outcomes on individuals’ mental health. The current work found a potential way to boost such behavior through positive comments. Positive comments affirming the information contained in the mental health message may contribute to reducing negative perceptions surrounding mental health, which is a key deterrent to seeking mental health treatment [12]. In addition using mental health services, sharing mental health information in social networks could help maximize the effects of social media mental health interventions and reach a larger audience, which in turn leads to positive and actionable health behaviors [35]. Future studies should explore the benefits of sharing health information on social media.

### 4.2. Design Applications

The current work provides empirical evidence supporting the notion that cues on social media significantly impact one’s trust regarding attitudes and behavioral intentions towards mental health, suggesting that source cue in health message persuasion is critical from a design perspective. As observed in the current work, health practitioners and researchers who aim to promote health messages should create authority-based source cues to signal their expertise to their target audience.

Further, health messages on social media is also a critical component for promoting effective and actionable health information since they can be shared online and reach a broader population. The findings of the current study are also consistent with prior work suggesting that different comments boost involvement with online content [35,48], indicating that health practitioners and researchers promoting health information using social media [49] should address negative user comments in a timely and professional matter, such as replying to user comments with accurate health information and contact information for local mental health services.

Summing up, this study has both theoretical and practical implications. This work provided empirical evidence for understanding the theoretical relationships between cues on social media and cognitive perceptions as a complex communication process. The proposed model extended the previous theoretical framework by building associations between cues and different types of cognitive perception. The current study also has practical implications which could provide insights for health researchers into understanding and designing social media mental health campaigns for college students in the US. However, individuals from different cultures or countries may have distinct reactions to mental health-related messages [50]. Therefore, more empirical studies in different cultures are needed for understanding mental health campaigns and prevention on social media.

### 4.3. Limitations and Future Directions

The current study has certain limitations. First of all, the Facebook posts did not employ a real Facebook page or group in an interactive setting. The screenshots of Facebook posts are different from the interactive and dynamic social media environment, and this may have influenced the results. Future studies should examine conditions in a real social media setting and evaluate the engagement of the participants on the social media platform. Second, the current study used cross-sectional data which only measures variables from a single time point and did not track longitudinal effects of the experiment. Future studies should use longitudinal design to collect data over time. Finally, the present study used an undergraduate student sample, which is appropriate since college students are a population that have been observed to be both heavy social media users and be vulnerable to different mental issues, such as depression [39]. However, we did not measure the mental health status of the study sample, warranting examination of these effects in an actual mental health intervention using a sample of individuals diagnosed with mental health disorders in the future. Future social mental health interventions could target individuals with mental health issues to increase their use of mental health services, and also target the general public to raise positive perceptions and increase their intentions towards using mental health services when they have the need. Future studies should also investigate the effects of source and comments in different cultures or countries and further explore the benefits of sharing health information on social media, which could expand the effects of social media campaigns and interventions.

## 5. Conclusions

The current work contributes to the growing issue of information source credibility in modern health communication, particularly in a mental health context. The present study explores the effects of cues on mental health information perceptions on social media and suggests that source and valence of comments are critical for promoting the seeking of mental health counseling. Specifically, we found that information source could influence cognitive trust, which ultimately impacts intentions to share mental health information on social media. We also found that positively-valenced comments significantly impact affective trust in the message, which ultimately impacts both intentions to share mental health information and to use mental health services, via its previous effects on attitudes.

## Figures and Tables

**Table 1:** Descriptive Statistics of Main Variables under Study.

Variable	Mean (SD)	Range
Cognitive trust	5.48 (1.72)	1–10
Affective trust	5.49 (1.75)	1–10
Attitudes toward Facebook posts of mental health information	4.57 (1.19)	1–7
Attitudes toward mental health services	5.60 (1.21)	1–7
Intention to share mental health information	2.45 (1.00)	1–5
Intention to use mental health services	3.79 (0.85)	1–5

**Table 2:** Summary of Model Fit.

Model	*χ*²	*df*	χ²/*df*	*RMSEA*	*SRMR*	*CFI*	*TLI*
Model of source and valence of comments on behavioral intentions	665.94	259	2.41	0.061	0.045	0.94	0.93

*df* = degree of freedom; *RMSEA* = root mean squared error of approximation; *CFI* = Comparative Fit Index; *TLI* = Tucker–Lewis index
